# MicroRNA-494 augments fibrotic transformation of human retinal pigment epithelial cells and targets p27 with cell-type specificity

**DOI:** 10.3389/fopht.2023.1168650

**Published:** 2023-04-14

**Authors:** Theodore Leng, Georgia Kamboj, Xiaoyun Sun, Heather Chang, Prisha Davda, Majesty Greer, Creed M. Stary

**Affiliations:** ^1^ Byers Eye Institute at Stanford, Stanford University School of Medicine, Palo Alto, CA, United States; ^2^ Department of Anesthesia, Pain and Perioperative Medicine, Stanford University School of Medicine, Stanford, CA, United States; ^3^ Howard University College of Medicine, Washington, DC, United States

**Keywords:** MiR-494, RPE, Müller cell, ERM, miR, miRNA, epiretinal membrane

## Abstract

Epiretinal membranes (ERMs) are the result of fibro-cellular proliferation that cause distortion and impairment of central vision. We hypothesized that select microRNAs (miRs) regulate retinal fibro-proliferation and ERM formation. Following IRB approval, a pilot study was performed in patients presenting for retina surgery with and without clinical ERMs. Total RNA was isolated from ERM tissue and controls from non-ERM vitreous and subjected to miR profiling *via* microarray analysis. MiR-494 was identified as the only miR selectively expressed at significantly greater levels, and *in silico* analysis identified p27 as a putative fibroproliferative gene target of miR-494. *In vitro* testing of miR-494 and p27 in fibrotic transformation was assessed in spontaneously immortalized human retinal pigment epithelial (RPE) and human Müller cell lines, stimulated to transform into a fibroproliferative state *via* transforming growth factor beta (TGFβ). Fibroproliferative transformation was characterized by *de novo* cellular expression of alpha smooth muscle actin (αSMA). In both RPE and Müller cells, both TGFβ and miR-494 mimic decreased p27 expression. In parallel experiments, transfection with p27 siRNA augmented TGFβ-induced αSMA expression, while only in RPE cells did co-transfection with miR-494 inhibitor decrease αSMA levels. These results demonstrate that miR-494 augments fibrotic transformation in both Müller cells and RPEs, however only in RPEs does miR-494 mediate fibrotic transformation *via* p27. As p27 is known to regulate cellular proliferation and differentiation, future studies should extend clinical testing of miR-494 and/or p27 as a potential novel non-surgical therapy for ERMs, as well as identify relevant miR-494 targets in Müller cells.

## Introduction

Epiretinal membrane (ERM) formation is a pathological, vitreoretinal proliferative process that results in distortion and impairment of central vision. ERMs are common, with a maximum prevalence of 35% during the 8^th^ decade of life (1). As such, ERMs remain the third most common indication for pars plana vitrectomy (2). Surgery can accelerate cataract progression and can be complicated by retinal tears and rhegmatogenous retinal detachment. Recurrence is common, up to 21% (2–4), therefore given the frequency of ERM and the singular invasive treatment option, further research into alternative treatments and prevention is warranted.

ERM formation is thought to be the result of transformation of retinal cells that proliferate into fibrous and contractile tissue over the macula. Retinal microglia cells secrete transforming growth factor beta (TGFβ) inducing trans-differentiation of Müller glial cells and retinal pigment epithelial cells (RPEs) to a myofibroblast phenotype (5). Müller cells, normally found in the inner limiting membrane (ILM), respond by growing processes toward the vitreous surface, breaking through the ILM and onto the macular surface (5, 6). There, they transform to an “activated” phenotype defined by upregulation of intermediate filaments such as nestin, vimentin, and glial fibrillary acidic protein (7, 8). RPEs similarly migrate through breaks in the retina and attach to the macular surface (9) after stimulation by TGFβ to undergo myofibroblastic differentiation (10). Another characteristic found in ERMs (5, 8) is immunoreactivity for alpha-smooth muscle actin (αSMA). Activated, myofibroblast-like αSMA+ cells function in producing extracellular matrix and collagen, which are key features in the transition from a cellular to a contractile fibrotic ERM. TGFβ promotes myofibroblast-like properties including upregulation of αSMA (11), however the molecular regulation underpinning TGFβ-mediated fibroproliferative transformation remains unknown.

MicroRNAs (miRs) are small, noncoding RNAs that modulate gene expression by acting as regulators of their messenger RNA (mRNA) targets (9, 10) Müller. The translational success of miR-based therapeutics in preclinical models has led to the successful development of pharmaceutical therapies for hepatitis C (12) and liver cancer (13), demonstrating that identification of relevant miR targets can be critical in the development of novel treatments for known diseases. Dysregulation in miR biology has been established as a central mechanism in several ocular diseases (9, 14). In the present study, we identified miR-494 as upregulated in clinical ERM samples relative to normal vitreous. Building on this observation, we therefore assessed in RPEs and Müller cells whether miR-494 plays a role in TFGβ-induced fibrotic transformation.

A recent study (15) in hepatocellular carcinoma demonstrated that elevated miR-494 expression resulted in increased cell proliferation *via* targeting and downregulating the cyclin-dependent kinase (CDK) inhibitor p27. Encoded by the CDKNB1 gene, p27 is a member of the kinase inhibitory protein (Kip) family (7) and functions as a tumor suppressor. CDKs regulate checkpoints that coordinate cell cycle transitions, and p27 regulates the cell cycle through inhibiting CDK and preventing the progression from G1 to S phase, thereby inhibiting proliferation (16). In normal retinal cells, p27 is expressed at high levels to inhibit cell proliferation, however in ERMs, p27 was observed to be downregulated (17, 18). Building on these observations, in the present study we assessed whether p27 plays a role in RPE and Müller cell fibrotic transformation, and in parallel we tested the hypothesis that miR-494 regulates fibrotic transformation by targeting p27.

## Methods

### Clinical ERM sampling and miR analysis

Human research protocols were approved by the Stanford University Internal Review Board (IRB) and this research conformed to the tenets of the Declaration of Helsinki. A pilot study was performed in patients presenting for vitreoretinal surgery with and without clinical ERMs. Samples of ERMs from patients and separate patients with normal vitreous for controls were collected aseptically during surgery in RNAase free 1.5 ml collection tubes and were immediately frozen with dry ice. Total RNA was isolated from ERM tissue and from non-ERM vitreous using TRIzol^®^ (ThermoFisher Scientific, Waltham, MA), and subjected to miR profiling *via* NanoString™ nCounter microarray analysis (#Hu v3, NanoString Technologies Inc., WA, USA) as we have done previously (19). Geometric mean was used to normalize the number of hybridized reads of each miR to spiked internal positive controls, and to the top 100 reads mean (nSolver Analysis Software 4.0, version 4.0.62, NanoString Technologies Inc.). The mean of negative controls plus 3 standard deviations (3σ) was used to determine significant difference (19). Patient demographics and exclusion criteria are presented in **Table 1**.

**Table 1:** Patient characteristics.

		Epiretinal Membrane Samples	Control (Vitreous Samples)
	N (%)	4 (66.7)	2 (33.3%)
	Mean Age (SD)	64.5 (1)	62 (11.3)
	Mean Visual Acuity (LogMAR (SD))	0.45 (0.2)	1.1 (1.0)
Gender	Female, n (%)	3 (75%)	0 (0%)
	Male, n (%)	1 (25%)	2 (100%)
Race	White, n (%)	3 (75%)	2 (100%)
	Black or African American, n (%)	0 (0%)	0 (0%)
	Asian, n (%)	1 (25%)	0 (0%)
	Other, n (%)	0 (0%)	0 (0%)
Ethnicity	Hispanic or Latino, n (%)	0 (0%)	2 (100%)
	Not Hispanic or Latino, n (%)	4 (100%)	0 (0%)

### Cell cultures

The human Müller cell line Moorfields/Institute of Ophthalmology- Müller 1 (MIO-M1) was obtained from the UCL Institute of Ophthalmology, London, UK (20). Immortalized human RPE cell cultures (ARPE-19) were sourced from ATCC (Manassas, VA), and obtained as a gift from the laboratory of Dr. Jeffrey Goldberg (Stanford University, Dept. Ophthalmology, Stanford, CA). Cells (25-33 passage) were seeded on 24-well plates at 1.5 X 10^5^ density in plating medium consisting of Eagle’s Minimal Essential Medium (Gibco, Grand Island, NY), supplemented with 10% fetal bovine serum (Hyclone, Logan, UT). Cultures were maintained at 37°C in a 5% CO_2_ incubator until confluent (3-4 days *in vitro*). Cell cultures were transformed into a fibroproliferative state *via* incubation with 10 ng/ml recombinant human TGFβ1 (#PHG9214, Thermofisher Scientific, Waltham, MA) for 72h. In parallel experiments Müller and RPE cultures were transfected 24h prior to TGFβ treatment with 50 pmol/well hsa-miR-494-3p mimic (#4464066, ThermoFisher Scientific), hsa-miR-494-3p inhibitor (#4464084, ThermoFisher Scientific) or mismatch control sequence (#4464058, ThermoFisher Scientific), using Lipofectamine 2000 (Invitrogen, Carlsbad, CA) according to the manufacturer’s protocol. Expression of miR-494 was confirmed by RT-qPCR, described below. Silencing of p27 was carried out by transfection with small interfering RNA to CDKN1B (50 pmol/well, #AM167808, ThermoFisher Scientific) using Lipofectamine 2000 (Invitrogen). The efficiency of the knockdown was assessed by quantification of p27 mRNA expression evaluated 24h after transfection *via* RT-qPCR (below). For all experiments 3-4 independent cultures were tested as replicates within each experiment, and the experiment was repeated 3-4 times using cells obtained from different dissections.

### Reverse transcription quantitative polymerase chain reaction (RT-qPCR)

Total RNA was isolated from cells with TRIzol^®^ (ThermoFisher Scientific). Reverse transcription was performed as previously described (21) using the TaqMan MicroRNA Reverse Transcription Kit for miR-494 and total RNA (Applied Biosystems, Foster City, CA). Predesigned primer/probes for PCR were obtained from ThermoFisher Scientific for hsa-miR-494 (#02252), U6 small nuclear RNA (U6, #01973), p27 mRNA (CDKN1B, #4400291) and glyceraldehyde 3-phosphate dehydrogenase mRNA (GAPDH, #4331182). PCR reactions were conducted as previously described (21) using the TaqMan^®^ Assay Kit (Applied Biosystems). Measurements for miR-494 were normalized to U6 (ΔCt), those for p27 were normalized to GAPDH. Comparisons were calculated as the inverse log of the ΔΔCT from controls (22).

### Immunoblotting

Immunoblotting was performed as previously described (23). Cells were harvested from 24 well plates with 4 wells per treatment group combined to provide sufficient protein for analysis. Equal amounts of protein (50μg *via* BCA Protein Assay, ThermoFisher Scientific) were then loaded and separated on a 4-12% polyacrylamide gel (Invitrogen), then electrotransferred to an Immobilon polyvinylidene fluoride membrane (EMD Millipore Corp. Burlington, MA, USA). Membranes were blocked and incubated overnight with primary antibody against αSMA (#19245, Cell Signaling Technology, Danvers, MA) and β-actin (1:1000, 926–42,210, LI-COR Bioscience, Lincoln, NE), washed and then incubated with 1:15,000 secondary antibodies (LI-COR Bioscience). Immunoreactive bands were visualized using the LI-COR Odyssey™ infrared imaging system according to the manufacturer’s protocol. Densitometric analysis of bands was performed blinded using Image-J software (v1.49b NIH).

### Fluorescent immunohistochemistry

*In situ* protein imaging was performed *via* fluorescent immunocytochemistry (IHC) as previously described (5). Cells were washed once with 0.1% PBS, fixed with ice-cold 4% paraformaldehyde for 10 min and then washed again three times with PBS. Afterward, the cells were permeabilized with 0.1% Triton-X in PBS for 10 min followed by blocking with 5% horse serum (HyClone) for 1h. Primary antibody against αSMA (#19245, Cell Signaling Technology) was diluted in blocking buffer and added to cells and then incubated overnight at 4°C for 24h. Next, cells were washed three times with PBS, followed by addition of secondary antibodies (1:10000) and the nuclear dye DAPI (4′,6-diamidino-2-phenylindole, ThermoFisher Scientific). Cells were imaged with an automated fluorescent microscope (Etaluma Lumascope 720) at 200x magnification. All imaging was performed using a fixed excitation intensity, exposure time, and gain, to minimize variability. No post-imaging processing was performed. An observer blinded to conditions quantified from 9 images per well the fluorescence of αSMA normalized to the fluorescence of DAPI.

### Statistics

Statistical difference was determined using one-way ANOVA with Tukey’s *post-hoc* comparison for experiments with >2 groups at a single time point or student’s t-test for comparison of two groups at a single time point. In all tests, *P*<0.05 was considered significant. Data reported are means ± SEM.

## Results

### Elevated expression of miR-494-3p in clinical ERM samples and *in vitro* after TGFβ stimulation

**Figure 1:**
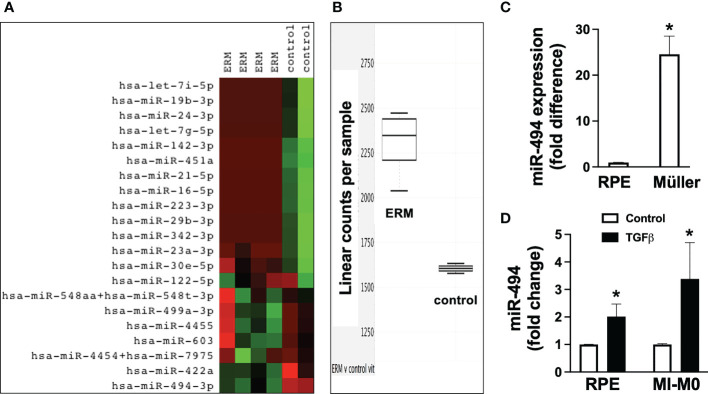
Expression of miR-494-3p in clinical epiretinal membrane (ERM) samples and *in vitro* after transforming growth factor beta (TGFβ) stimulation in human Müller cell line and human retinal pigment epithelial cell (RPE) cell lines. Heat map **(A)** of miR expression profiles and RT-qPCR quantification **(B)** of miR-494-3p from 4 ERM samples were compared with profiles from 2 vitreous samples from eyes without ERMs. Expression of miR-494 in human Müller cell line and human RPE cell line at baseline **(C)** and 72h after incubation with TGFβ **(D)**. Mean ± SEM, * p<0.05 versus RPE or TGFβ versus control condition.

In order to identify potential miRs that could be dysregulated in ERM formation for potential therapeutic targeting, expression profiles from 4 ERM samples were compared with profiles from 2 vitreous samples from eyes without ERMs. Heat map (**Figure 1A**) visually identified miR-494-3p as the only miR selectively expressed at significantly greater levels in all ERM tissues compared with control from a panel of 828 human miRs, and validated as significantly elevated (**Figure 1B**; p=0.002; CI: 1.23-1.66). At baseline, the human Müller cell line expressed significantly (p<0.05) greater miR-494 than the human RPE cell line (**Figure 1C**), however both cell types responded with significantly (p<0.05) increased miR-494 expression 24h after incubation with TGFβ (**Figure 1D**). These results suggest that miR-494 could be a viable therapeutic target to modulate or mitigate clinical ERM formation.

### TGFβ stimulation induces αSMA expression that can be suppressed by miR-494 inhibition

**Figure 2:**
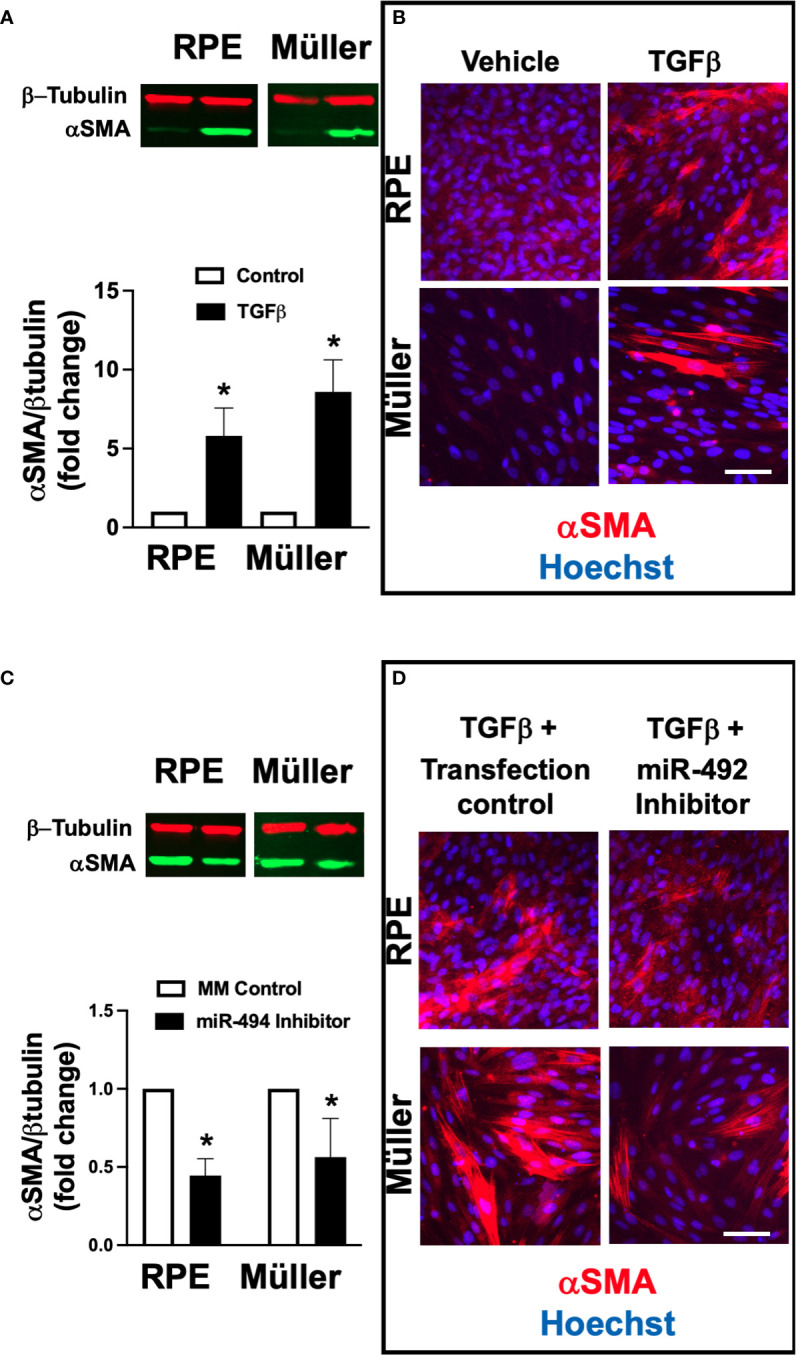
Effect of alpha smooth muscle actin (αSMA) expression after TGFβ stimulation with and without miR-494 inhibition in human Müller cell line and human RPE cell lines. **(A)** Quantification of αSMA in human RPE and Müller cell lines by immunoblot (representative blots above) with and without 72h TGFβ treatment. **(B)** Representative immunofluorescent images of αSMA (red) in human RPE and Müller cell lines with and without 72h TGFβ treatment and counterstained with the nuclear dye 4′,6-diamidino-2-phenylindole (DAPI, blue). **(C)** Quantification of αSMA in human RPE and Müller cell lines by immunoblot (representative blots above) after 72h TGFβ treatment with and without pre-treatment and co-incubation with miR-494-3p inhibitor. **(D)** Representative immunofluorescent images of αSMA (red) in human RPE and Müller cell lines counterstained with the nuclear dye DAPI (blue) after 72h TGFβ treatment with and without pre-treatment and co-incubation with miR-494-3p inhibitor. Mean ± SEM, * p<0.05 TGFβ versus control condition.

To assess the potential mechanistic role of miR-494 in ERM formation we utilized an established *in vitro* model of cellular fibrotic transformation in human RPE and Müller cell lines. Incubation with 10 ng/ml recombinant human TGFβ1 for 72h resulted in significant (p<0.05) increases in αSMA expression in both human RPE and Müller cell lines (**Figures 2A, B**). Treatment with miR-494 inhibitor significantly decreased miR-494 expression to <5% compared to mismatch control sequence in both cell types. MiR-494 inhibitor significantly (p<0.05) reduced αSMA expression in both RPE and Müller cell lines (**Figures 2C, D**) suggesting a central role for miR-494 in cellular fibrotic transformation independent of cell type.

### The CDK inhibitor p27 regulates fibrotic transformation in both RPE and Müller cell lines

**Figure 3:**
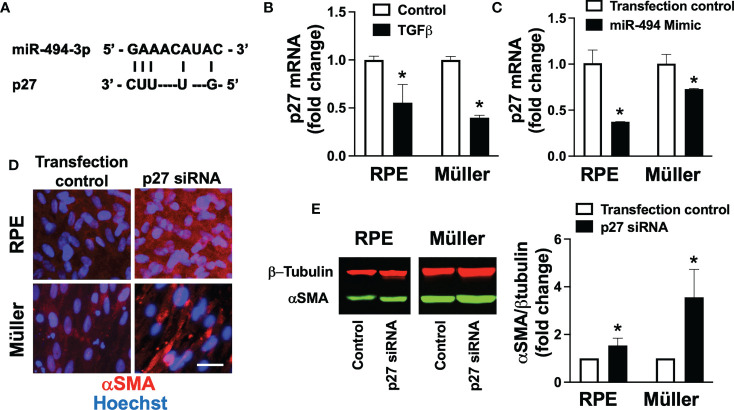
p27 in TGFβ-induced fibrotic transformation. **(A)** Diagram of reverse complementary miR-494 and p27 binding sites. **(B)** Quantification of p27 mRNA in human RPE and Müller cell lines with and without TGFβ treatment. **(C)** Quantification of p27 mRNA in human RPE and Müller cell lines with and without miR-494 mimic treatment. **(D)** Representative immunofluorescent images of αSMA (red) in human RPE and Müller cell lines with DAPI (blue) after 72h TGFβ treatment with and without pre-treatment and co-incubation with p27 small interfering RNA (siRNA). **(E)** Quantification of αSMA in human RPE and Müller cell lines by immunoblot (representative blots left) after 72h TGFβ treatment with and without pre-treatment and co-incubation with p27 siRNA. Mean ± SEM, * p<0.05 versus control condition.

In order to identify potential downstream therapeutic targets of miR-494 we performed an *in silico* reverse complementarity analysis and identified p27 as a fibroproliferative gene target of miR-494 (**Figure 3A**). Incubation with TGFβ resulted in significantly (p<0.05) decreased p27 mRNA expression in both human RPE and Müller cell lines (**Figure 3B**). Treatment with miR-494 mimic significantly increased levels of miR-494 expression (>2000 fold in both cell types) and resulted in a significant reduction in p27 mRNA expression in both cell types (**Figure 3C**). Transfection with p27 siRNA significantly (p<0.05) increased αSMA in both cell types (**Figures 3D, E**), indicating that p27 suppression alone can induce fibrotic transformation in both cell types.

### miR-494 modulates fibrotic transformation *via* targeting of p27 only in RPE cells

**Figure 4:**
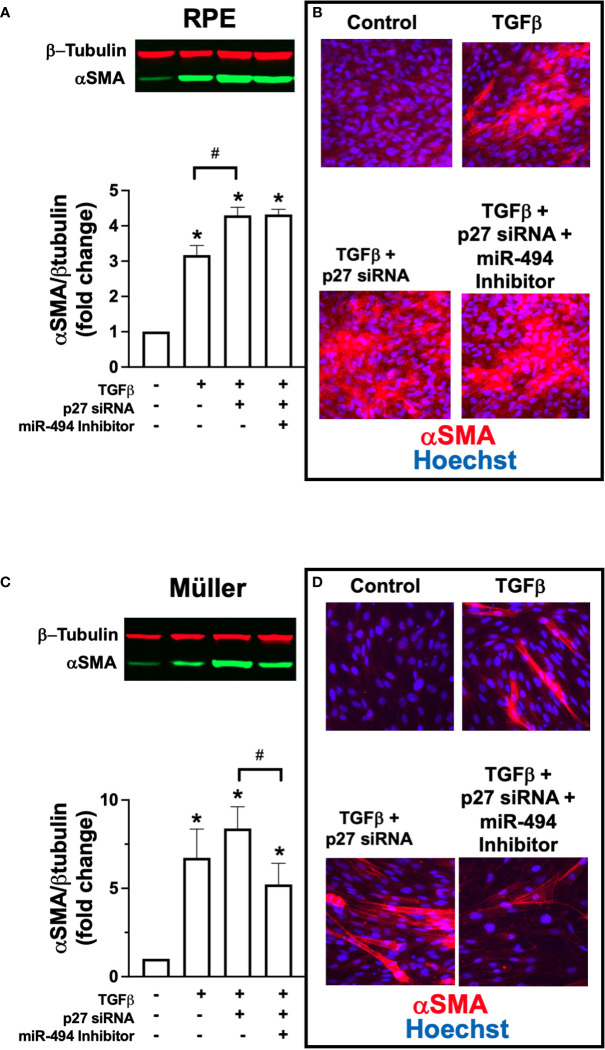
Effect of co-treatment with p27 siRNA and miR-494 inhibitor on TGFβ-induced αSMA expression. **(A)** Quantification of αSMA in RPE cells by immunoblot (representative blots above) after 72h TGFβ treatment with and without pre-treatment and co-incubation with miR-494 inhibitor with or without p27 siRNA co-transfection. **(B)** Representative immunofluorescent images of αSMA (red) in RPE cells counterstained with DAPI (blue) after 72h TGFβ treatment pre-treatment and co-incubation with miR-494 inhibitor with or without p27 siRNA co-transfection. **(C)** Quantification of αSMA in Müller cells by immunoblot (representative blots above) after 72h TGFβ treatment with and without pre-treatment and co-incubation with miR-494 inhibitor with or without p27 siRNA co-transfection. **(D)** Representative immunofluorescent images of αSMA (red) in Müller cells counterstained with DAPI (blue) after 72h TGFβ treatment pre-treatment and co-incubation with miR-494 inhibitor with or without p27 siRNA co-transfection. Mean ± SEM, * p<0.05 versus control condition. ^#^ p<0.05 between select conditions.

Finally, to investigate direct targeting of p27 by miR-494 we co-transfected each human cell line with p27 siRNA and miR-494 inhibitor. In RPE cells, co-treatment with p27 siRNA resulted in an additive effect with TGFβ-induced αSMA expression (**Figures 4A, B**). However, pre-treatment and co-incubation with miR-494 inhibitor had no effect on augmented αSMA expression with p27 siRNA (**Figures 4A, B**). As miR-494 inhibition failed to provide a protective effect against fibrotic transformation with p27 inhibition, this finding suggests that p27 is a primary mechanistic target of miR-494 in RPE cell fibrotic transformation. Conversely, in Müller cells, pre-treatment and co-incubation with miR-494 inhibitor with p27 siRNA resulted in a significant decrease in TGFβ-induced αSMA expression versus p27 siRNA (**Figures 4C, D**), indicating that miR-494 inhibition could provide protection against fibrotic transformation *via* alternative mechanisms independent of p27.

## Discussion

**Figure 5:**
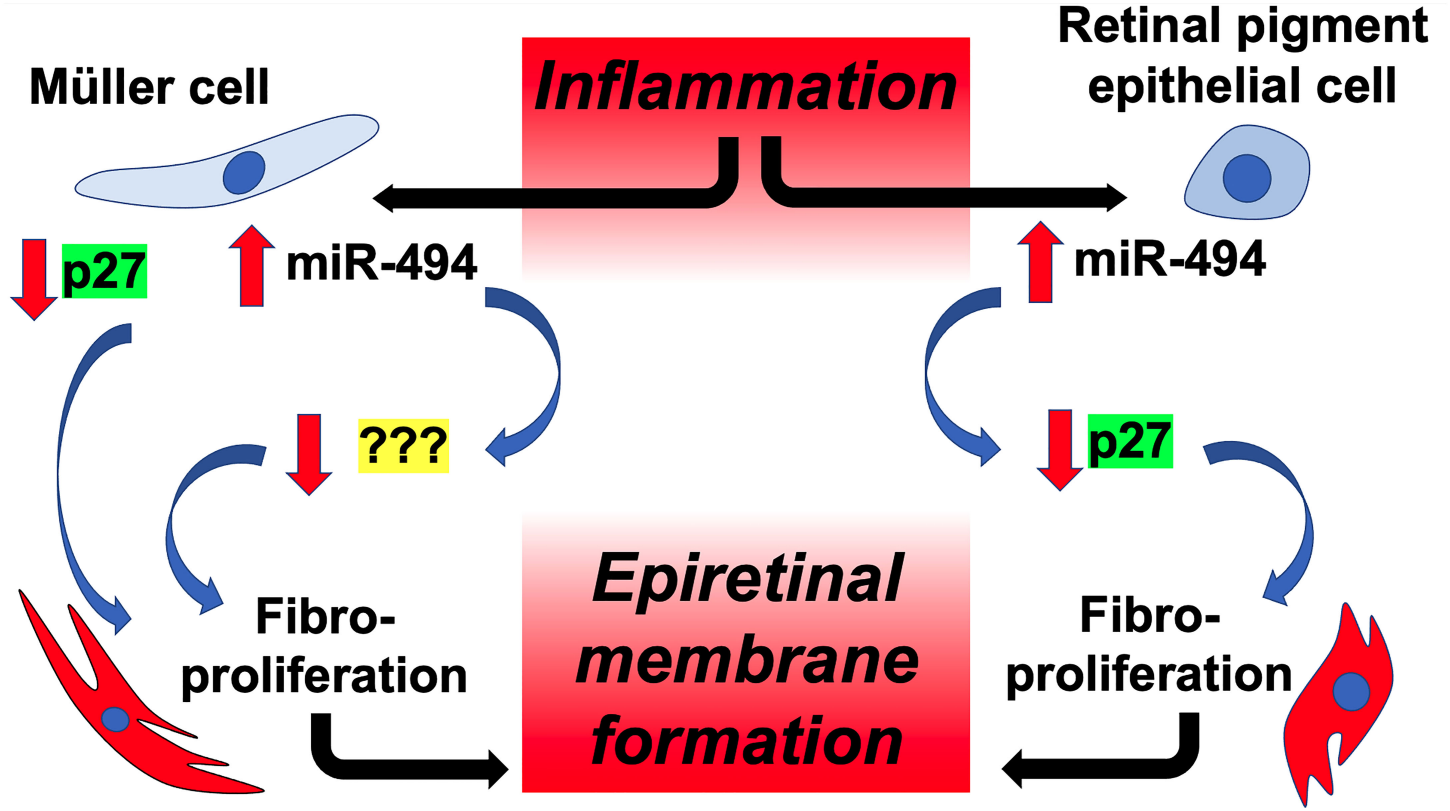
Proposed model of cell-type specific targeting of p27 by miR-494 in ERM fibrotic transformation. Microglial-mediated inflammation triggers elevations in miR-494 expression in both Müller cells and RPEs, leading to downstream fibrotic transformation in both cell types, and ultimately to ERM formation. Although decreases in p27 play a central role in fibrotic transformation in both cell types, only in RPE cells is reduced expression of p27 due to direct targeting by miR-494.

Based on the results of the present study, a proposed cell-type specific model of miR-494-mediated fibrotic transformation in Müller and RPE cells is presented in **Figure 5**. We utilized TGFβ to induce fibrotic transformation in both human Müller and RPE cell lines to model clinical ERM formation. In both cell types, incubation with TGFβ resulted in *de novo* expression of αSMA, a phenotypic marker for fibrotic transformation (24). In the present study αSMA was selected as a fibrotic marker as it is expressed at high levels in both RPE and Müller cells allowing for intercellular comparisons of fibrotic change, versus alternative fibrotic markers associated ERMs such as glial-fibrillary acidic protein or vimentin (25) that are instead derived primarily from Müller cells. Increased αSMA expression provides myofibroblasts with stronger contractile activity and is more commonly seen in ERMs causing tractional complications (5, 26). Identifying molecular targets in αSMA induction and regulation of ERM fibrotic transformation could provide novel non-invasive therapies for ERM.

Research demonstrating a central role for miRs in molecular regulation of retina disease is rapidly expanding (27). Several miRs have been found to be dysregulated in rodent models of age-related macular degeneration such as miR-146a, miR-17, miR-125b, and miR-155 (28), linked to cellular processes such as inflammation, angiogenesis, apoptosis, and phagocytosis. Other miRs have been shown to be linked to progression of diabetic retinopathy, functioning as markers of disease progression (29). In the present study we identified miR-494 as significantly unregulated in human ERM samples relative to healthy control vitreous, suggesting a role for miR-494 in this process. The study was limited in size and scope and should be validated in larger and more diverse cohorts, at multiple institutions. However, to support these preliminary observations for clinical utility of miR-494 as a target for ERM we demonstrated in both RPEs and Müller cells that miR-494 mimic functionally induced αSMA expression, while miR-494 inhibitor had the opposite effect of preventing TGFβ-induced elevations in αSMA. In support of our present observations, a recent relevant study (30) demonstrated elevations in miR-494 contributed to cell proliferation and disease progression in retinoblastoma (5). In other organ systems prior studies have identified a central role for miR-494 in epithelial to mesenchymal transition (EMT), a process similar to retinal fibrotic transformation, including the genitourinary (31–33), gastrointestinal tract (34–36) and pulmonary systems (37). However, the biologic effects of miRs can be tissue-type dependent, and miR-494 has been shown to behave either as an oncogene or a tumor suppressor depending on the organ affected (15). For example, in colorectal cancer (38) and in non-small cell lung cancer (5) miR-494 functions as an oncogene to promotes cell proliferation, while in gastric carcinoma (29) and breast cancer (39) miR-494 reduces cellular transformation and proliferation. Further pre-clinical studies in animal models of ERM are warranted to more accurately assess miR-494 as a functional therapeutic target.

In the present study we also identified a central role for p27 in fibrotic transformation in both cell lines. Mitogenic factors cause loss of p27, whereas p27 levels and/or activity increase in response to differentiation signals. Mice with p27 knockout develop multiorgan hyperplasia and pituitary tumors (40). Whereas p27 mRNA levels are constant throughout the cell cycle, p27 protein levels are high in quiescent cells and decrease during G_1_ phase, reaching the lowest point in S phase (41). In the present study TGFβ resulted in a reduction in p27 mRNA, suggesting disruption of cellular p27 homeostasis and cell cycle regulation. Supporting our observations, a prior study (17) proposed that downregulation of p27 could be a contributing cause to Müller cell proliferation. Although nuclear p27 has been found to be a tumor suppressor, cytoplasmic p27 can act as an oncogene and contribute to cancer metastasis (42). One limitation of the present study is that we did not account for competing effects between nuclear and cytoplasmic fractions by determining sub-cellular changes in p27 expression. Future studies should explore whether sub-cellular p27 targeting could confer additional advantages over non-specific p27 targeting.

In addition to tissue-type specific effects of miR biology, miRs also exhibit cell-type specific expression patterns and targeting, including in the retina (9). For example, single cell RNA sequencing has revealed that certain miR species are expressed in all retinal cell types, while others are cell type-specific (9). In the present study we observed a greater baseline expression of miR-494 in human Müller cell line compared with human RPE cell line, and a differential response between cell types to fibrotic transformation with miR-494 mimic and p27 siRNA. In order to investigate whether cell-type specificity of miR-494 targeting of p27, co-transfection experiments of p27 siRNA with miR-494 inhibitor revealed that only in RPE cells is fibrotic transformation mediated by miR-494 *via* p27 targeting (**Figure 5**). Differential gene targeting by an individual miR between cell types could be the result of differential post-transcriptional chemical modifications on target mRNAs, including RNA methylation and acetylation, thereby modulating miR binding affinity (43). Alternatively, or in combination, alterations in RNA-binding protein expression could also account for differences in miR biological activity and gene targeting (44). In addition to exploring these mechanisms, additional studies should build on our observations to also identify the relevant miR-494 targets that regulate downstream fibrotic transformation in Müller cells. Alternative targets of miR-494 that have been identified in other organ systems that could potentially regulate retinal fibrotic transformation in Müller cells include phosphatase and tensin homolog (31), prolifin-2 (34), WD repeat and HMG-box DNA binding protein 1 (35), syndecan 1 (36), cullin 4A (33), suppression of cytokine signaling 6 (32), YTH N6-methyladenosine RNA binding protein 2 (37) and ten eleven translocation 1 (45).

Independent of miR-494, we also observed that p27 played a central role in fibrotic transformation in both cell types (**Figure 5**). Future studies also investigating CDK inhibition and/or other cell cycle regulators as putative therapeutic targets in ERM pathogenesis are therefore warranted in the search for novel non-surgical ERM therapies. One limitation of the present study is that experiments were limited to human cell lines. While human *in vitro* studies provide species specific and cell-type specific mechanistic investigations, future studies could implement a recently developed *in vivo* murine model of ERM formation (46) which would provide advantages of maintaining the retinal intercellular milieu as well as allowing for genetic manipulation.

## Data availability statement

The raw data supporting the conclusions of this article will be made available by the authors, without undue reservation.

## Ethics statement

The studies involving human participants were reviewed and approved by Stanford University Internal Review Board. The patients/participants provided their written informed consent to participate in this study.

## Author contributions

TL helped conceive the project and edited the manuscript, GK performed experiments and data analysis, XS performed experiments and data analysis, HC performed experiments and data analysis, PD performed experiments and data analysis, MG performed experiments and data analysis, CS helped conceive the project, and assembled and edited the manuscript. All authors contributed to the article and approved the submitted version.
